# Quantitative ^1^H NMR metabolomics reveals extensive metabolic reprogramming of primary and secondary metabolism in elicitor-treated opium poppy cell cultures

**DOI:** 10.1186/1471-2229-8-5

**Published:** 2008-01-22

**Authors:** Katherine G Zulak, Aalim M Weljie, Hans J Vogel, Peter J Facchini

**Affiliations:** 1 Department of Biological Sciences, University of Calgary, Calgary, Alberta, T2N 1N4, Canada

## Abstract

**Background:**

Opium poppy (*Papaver somniferum*) produces a diverse array of bioactive benzylisoquinoline alkaloids and has emerged as a model system to study plant alkaloid metabolism. The plant is cultivated as the only commercial source of the narcotic analgesics morphine and codeine, but also produces many other alkaloids including the antimicrobial agent sanguinarine. Modulations in plant secondary metabolism as a result of environmental perturbations are often associated with the altered regulation of other metabolic pathways. As a key component of our functional genomics platform for opium poppy we have used proton nuclear magnetic resonance (^1^H NMR) metabolomics to investigate the interplay between primary and secondary metabolism in cultured opium poppy cells treated with a fungal elicitor.

**Results:**

Metabolite fingerprinting and compound-specific profiling showed the extensive reprogramming of primary metabolic pathways in association with the induction of alkaloid biosynthesis in response to elicitor treatment. Using Chenomx NMR Suite v. 4.6, a software package capable of identifying and quantifying individual compounds based on their respective signature spectra, the levels of 42 diverse metabolites were monitored over a 100-hour time course in control and elicitor-treated opium poppy cell cultures. Overall, detectable and dynamic changes in the metabolome of elicitor-treated cells, especially in cellular pools of carbohydrates, organic acids and non-protein amino acids were detected within 5 hours after elicitor treatment. The metabolome of control cultures also showed substantial modulations 80 hours after the start of the time course, particularly in the levels of amino acids and phospholipid pathway intermediates. Specific flux modulations were detected throughout primary metabolism, including glycolysis, the tricarboxylic acid cycle, nitrogen assimilation, phospholipid/fatty acid synthesis and the shikimate pathway, all of which generate secondary metabolic precursors.

**Conclusion:**

The response of cell cultures to elicitor treatment involves the extensive reprogramming of primary and secondary metabolism, and associated cofactor biosynthetic pathways. A high-resolution map of the extensive reprogramming of primary and secondary metabolism in elicitor-treated opium poppy cell cultures is provided.

## Background

Opium poppy (*Papaver somniferum*) is the world's oldest medicinal plant and produces several pharmaceutically important benzylisoquinoline alkaloids, including the analgesics morphine and codeine, the muscle relaxant and vasodilator papaverine, the antineoplastic drug noscapine and the antimicrobial agent sanguinarine. Benzylisoquinoline alkaloid biosynthesis in opium poppy begins with the condensation of dopamine and 4-hydroxyphenylacetaldehyde by norcoclaurine synthase (NCS) to yield (*S*)-norcoclaurine [1,2]. Several cDNAs encoding the multitude of enzymes that subsequently convert (*S*)-norcoclaurine to more than 80 benzylisoquinoline alkaloids in opium poppy have been isolated [3]. Opium poppy can be considered a model system to investigate the biology of plant alkaloid metabolism.

Alkaloid biosynthesis and accumulation are constitutive, organ- and cell type-specific processes in the plant. Morphine, noscapine and papaverine are generally the most abundant alkaloids in aerial organs, whereas sanguinarine typically accumulates in roots [4]. Alkaloid biosynthetic enzymes and cognate transcripts have been specifically localized to sieve elements of the phloem and associated companion cells, respectively [5,6]. In contrast, opium poppy cell cultures do not constitutively accumulate alkaloids, and produce only sanguinarine in response to treatment with specific fungal elicitors [7]. Elicitor-induced sanguinarine biosynthesis in opium poppy cell cultures provides a platform to definitively characterize the environmental induction of alkaloid and other secondary metabolic pathways under precisely controlled conditions. Moreover, the establishment of an extensive array of genomics resources, including expressed sequence tags (ESTs) and DNA microarrays [8], for opium poppy plants and cell cultures has also accelerated the development of a systems biology approach to discover new alkaloid biosynthetic genes and relevant biological processes.

Alterations in metabolite profile can be considered the ultimate cellular consequence of environmental perturbations. Together with other relatively unbiased and high-throughput technologies, metabolomics has facilitated an improved understanding of cellular responses to environmental change. Reports of metabolite profiling in the context of defence-related plant secondary metabolism, although rare, include the analysis of elicitor-treated *Medicago truncatula *cell cultures using gas chromatography-mass spectrometry (GC-MS) [9], carotenoid profiling using matrix-assisted laser desorption ionization time-of-flight mass spectrometry (MALDI-TOF) [10], and studies of phenylpropanoid and monoterpenoid indole alkaloid biosynthesis in phytoplasma-infected *Catharanthus roseus *leaves [11], caffeic acid and terpenoid metabolism in tobacco mosaic virus infected tobacco cells [12], and hydroxycinnamates and glucosinolates accumulation in methyl jasmonate (MeJA)-treated *Brassica rapa *leaves [13] using proton nuclear magnetic resonance (^1^H NMR). Although the use of ^1^H NMR for metabolite fingerprinting in the biomedical field is well established, reports of its application to plants are less extensive [14].

We have previously used Fourier transform ion cyclotron resonance-mass spectrometry (FT-ICR-MS) to show that substantial modulations in the metabolome of elicitor-treated opium poppy cell cultures are accompanied by major alterations in the transcriptome [8]. Although FT-ICR-MS analysis resolved 992 analytes, including several alkaloid pathway intermediates and products, only a few compounds could be identified solely on the basis of mass and corresponding molecular formula. A complementary technology is required to further characterize the specific alterations that occur in the metabolome of opium poppy cell cultures in response to elicitor treatment.

The advantages of nuclear magnetic resonance (NMR) spectroscopy over MS for metabolomics applications include the relative ease of sample preparation, non-destructive analysis, the potential to identify a broad range of compounds, an enhanced capacity for definitive compound identification, and the provision of structural information for unknown compounds [14,15]. Several plant studies have used NMR-based metabolite fingerprinting to catalogue general changes in the metabolome without identifying specific metabolites. The profiling of specific compounds using the NMR spectra of relatively crude plant extracts is hampered by several problems including spectral complexity, overlapping resonance peaks, and the lack of a comprehensive spectral library of standard compounds. In this paper, we report the application of ^1^H NMR to characterize the metabolome of elicitor-induced opium poppy cell cultures. We use a novel tool, Chenomx NMR Suite v. 4.6, to overcome many prior limitations in the analysis of ^1^H-NMR spectra [16]. The software package includes a metabolite library constructed by chemically modeling compounds of interest using their peak center and *J*-coupling information. This library was used to analyze the spectra of sample extracts and create mathematical models for detected metabolites in a cumulative manner. The chemometric strategies of principal component analysis (PCA) and orthogonal partial least-squares-discriminant analysis (OPLS-DA) were used to extract and display the systematic variation in the datasets. Our results show that the induction of secondary metabolism in response to elicitor treatment is accompanied by an extensive reprogramming of specific primary pathways.

## Results

### Global metabolite profiling of the elicitation response

**Figure 1:**
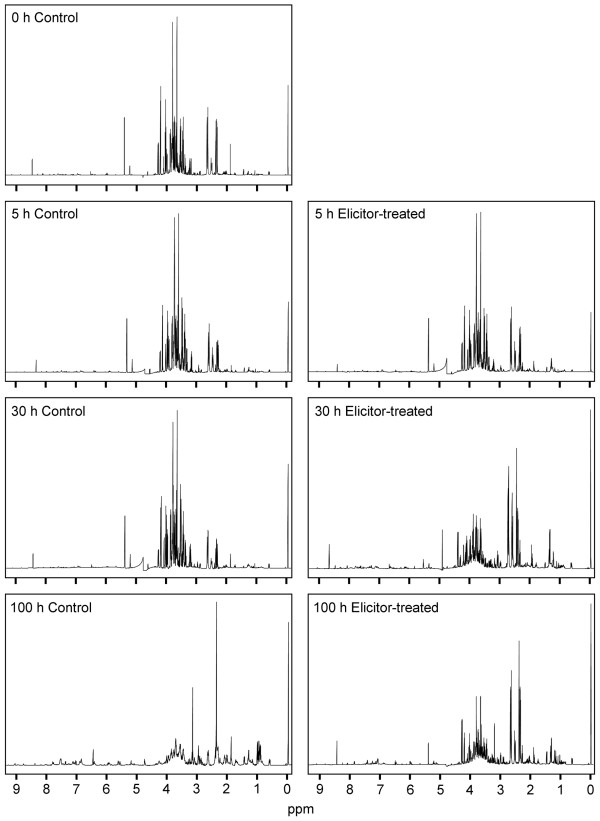
**^1^H NMR spectra of D_2_O extracts from control and elicitor-treated opium poppy cell culture collected 0, 5, 30 and 100 h post-elicitation**. 2,2-Dimethyl-2-silapentane-5-sulfonate (DSS) was used as an internal standard. The peak height of DSS, which was set at 0 ppm, is equivalent for all spectra.

**Figure 2:**
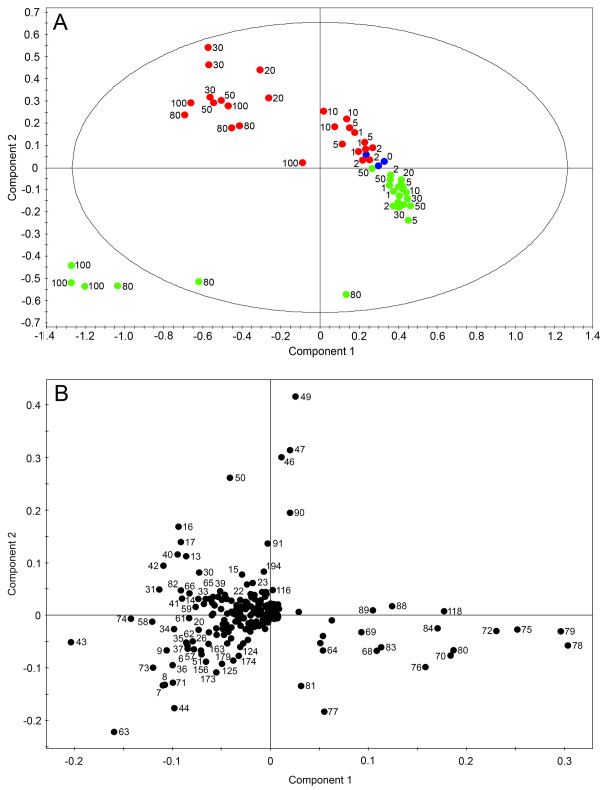
**Scores (A) and corresponding loadings plot (B) of principal component analysis (PCA) on ^1^H NMR spectra for D_2_O extracts of control (green) and elicitor-treated (red) opium poppy cell cultures collected at different time points post-elicitation**. The ellipse in A represents the Hotelling with 95% confidence. Numbers beside data point on the loadings plot correspond to specific bins used in the analysis.

Aqueous extracts of control and elicitor-treated cell suspension cultures of opium poppy were analyzed in D_2_O by ^1^H NMR. Figure 1 shows typical spectra obtained at 0, 5, 30 and 100 h post-elicitation. The most substantial differences in the NMR spectra occurred 30 h after elicitor treatment in the region corresponding to sugars (3.0–4.5 ppm). Few differences were observed in the spectra for 30 h-control samples, however the 100 h-control spectra were substantially different from elicitor-treated spectra at the same time point, especially the aromatic (6.5–8.0 ppm) and aliphatic amino acid/organic acid (0.5–1.5 ppm) regions. Principal component analysis (PCA) was performed on three independent biological replicates of each time-point for both control and elicitor-treated cells (Figure 2A). The first principal component (PC1) separated the samples with respect to time and accounted for 65.6% of the variance within the data. The second principal component (PC2) separated the samples into control and elicited-treated groups and accounted for 17.4% of the variance.

The PCA scores plot (Figure 2A) shows rapid and dynamic changes in the metabolome of cultured opium poppy cells in response to elicitor treatment that are not apparent in control cell cultures. Samples collected 20 to 100 h after elicitor treatment diverged significantly from earlier time points. In contrast, only the 80 and 100 h control samples diverged from those collected at earlier control time points. A corresponding loadings plot shows the spectral regions (i.e. bins) responsible for the variation among samples (Figure 2B). Samples on the PCA scores plot (Figure 2A) and bins on the loadings plot (Figure 2B) that fall within the same quadrant represent specific NMR spectral regions with peaks that are higher in those samples, compared with all others, and contribute most extensively to the variance at different time points and between control and elicited-treated cells. Specific metabolites were identified within each numbered [see Additional file 1]. It is important to note that some bins contained more than one metabolite; thus, the metabolite directly responsible for the observed variance could not be unambiguously assigned without compound-specific profiling. Carbohydrates such as glucose, fructose and sucrose were more abundant in the 0–50 h control cultures and were most responsible for the variance at different time points in both control and elicitor-treated cells. Malate, citrate, threonine, and γ-aminobutyric acid (GABA) were among the metabolites more abundant in cells 20–100 h post-elicitation, compared with controls. Glutamine, 2-oxoglutarate, choline, and amino acids, such as leucine, valine, isoleucine, tyrosine and asparagine were found at higher levels in control extracts at 80 and 100 h, and discriminated these samples from elicitor-treated extracts at these time points.

**Figure 3:**
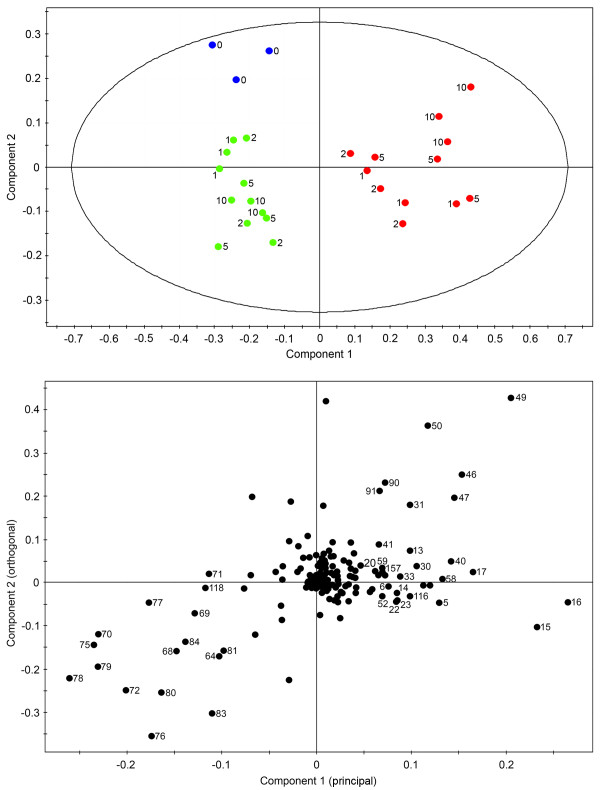
**Scores (A) and corresponding loadings plot (B) of orthogonal partial least-squares-discriminant analysis (OPLS-DA) on ^1^H NMR spectra for D_2_O extracts of control (green) and elicitor-treated (red) opium poppy cell cultures collected at 0, 1, 2, 5, and 10 h post-elicitation**. The ellipse in A represents the Hotelling with 95% confidence. Numbers beside data point on the loadings plot correspond to specific bins used in the analysis.

**Figure 4:**
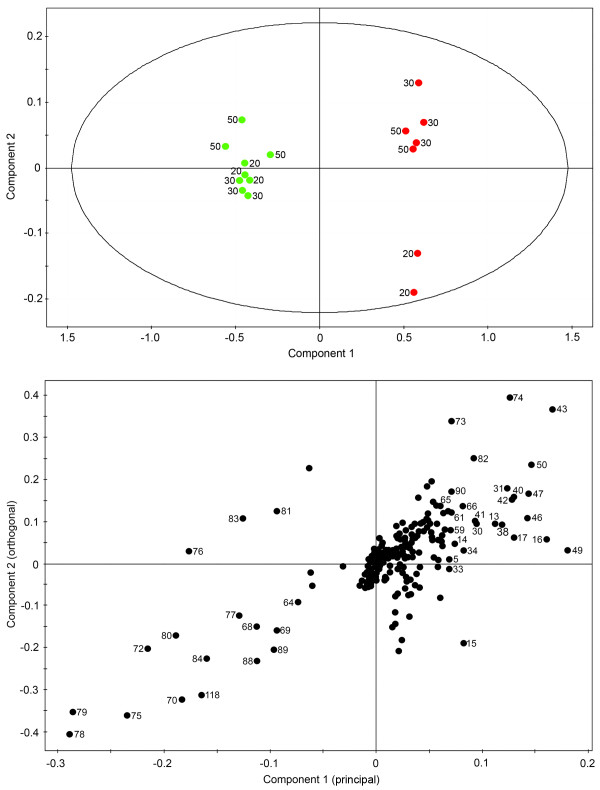
**Scores (A) and corresponding loadings plot (B) of orthogonal partial least-squares-discriminant analysis (OPLS-DA) on ^1^H NMR spectra for D_2_O extracts of control (green) and elicitor-treated (red) opium poppy cell cultures collected at 20, 30 and 50 h post-elicitation**. The ellipse in A represents the Hotelling with 95% confidence. Numbers beside data point on the loadings plot correspond to specific bins used in the analysis.

**Figure 5:**
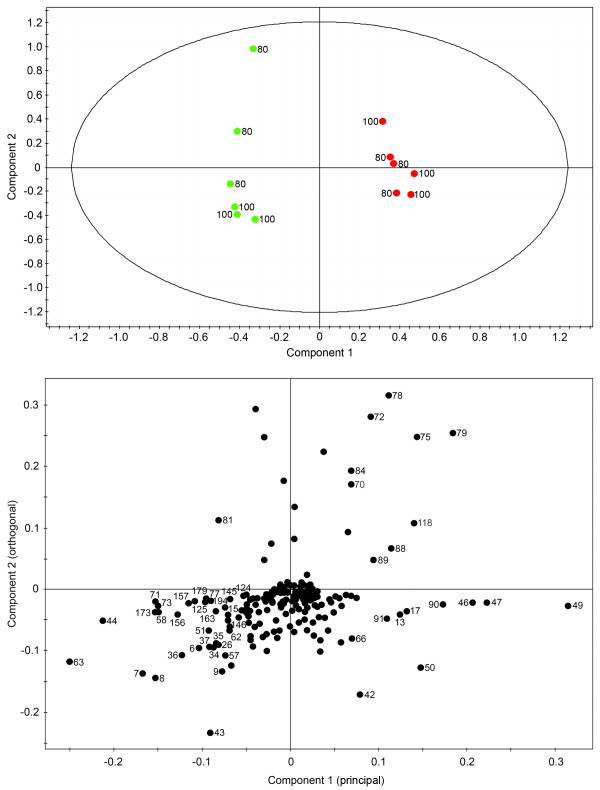
**Scores (A) and corresponding loadings plot (B) of orthogonal partial least-squares-discriminant analysis (OPLS-DA) on ^1^H NMR spectra for D_2_O extracts of control (green) and elicitor-treated (red) opium poppy cell cultures collected at 80 and 100 h post-elicitation**. The ellipse in A represents the Hotelling with 95% confidence. Numbers beside data point on the loadings plot correspond to specific bins used in the analysis.

Orthogonal partial least-squares-discriminant analysis (OPLS-DA) was performed on three groups of time-points: 0–10 h, 20–50 h and 80–100 h. This algorithm reveals more subtle changes in the occurrence and concentration of specific metabolites by focusing on compounds responsible for the discrimination between two classes (i.e. control and elicitor-treated samples). Modulations in metabolite profile within these three time-point groups were predominantly responsible for the discrimination between control and elicitor-treated cell cultures according to the PCA (Figure 2A). OPLS-DA on the 0–10 h time points showed a clear separation of control and elicitor-treated samples along the principal component (Figure 3). Unlike PCA, the bins in the OPLS-DA are assigned a variable importance, with higher numbers corresponding to bins that contributed more substantially to the explained variance between control and elicitor-treated cells at any given time point [see Additional file 1]. Citrate, malate, caprylate and threonine were the detectable metabolites that increased in abundance between 0–10 h in elicitor-treated cells, whereas the levels of sugars decreased. Similarly, changes in the levels of specific metabolites between 20–50 h were due mainly to an increase in the cellular pools of organic acids, GABA, threonine and several unidentified compounds, and decreased levels of sugars (Figure 4). In elicitor-treated cells, 20 h samples showed a substantial deviation from those collected at 30 and 50 h indicating that a major alteration in the metabolome occurred approximately 30 h post-elicitation. In contrast all time points clustered together in control samples. In 80 and 100 h extracts, organic acids, sugars and several unidentified compounds are nearly absent in controls, whereas choline, glutamine and other amino acids, and 2-oxoglutarate increased (Figure 5). At these time points, elicitor-treated samples clustered more closely than controls.

### Metabolite-specific profiling

**Figure 6:**
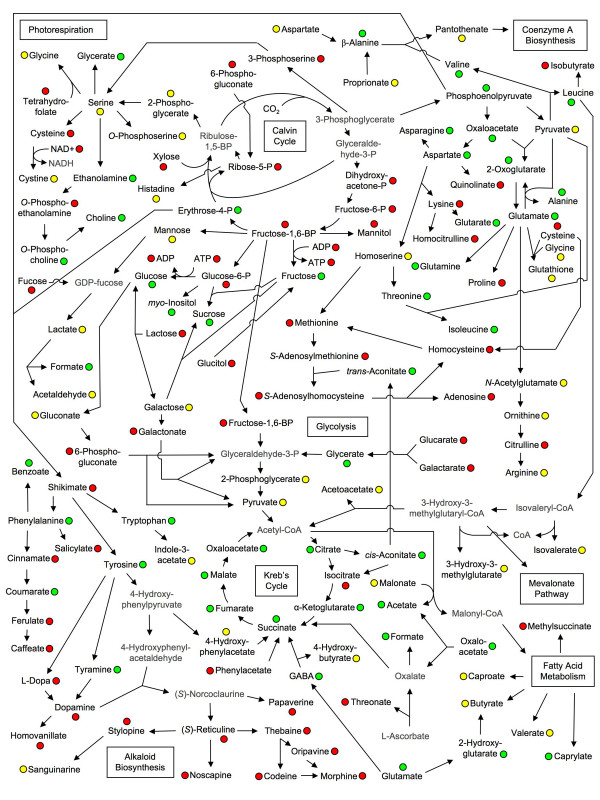
**Metabolite linkage map representing primary and secondary plant metabolism in opium poppy**. The circles associated with each metabolite indicate whether the metabolite was detected (green), not detected (red) or masked (yellow). Data could not be obtained for metabolites shown in grey because information regarding their standard ^1^H NMR spectra was not available.

**Figure 7:**
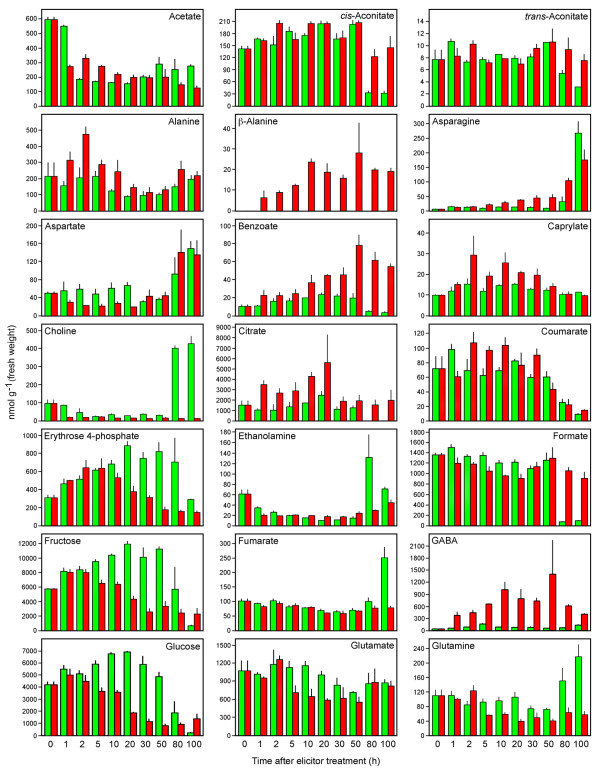
**Quantification of identified metabolites (acetate to glutamine, alphabetically) in control (green) and elicitor-treated (red) opium poppy cell cultures at different time points post-elicitation**. Data are given as means ± SEM, which were calculated using three biological replicates. Quantification was achieved using Chenomx NMR Suite v. 4.6 with DSS as the internal standard.

**Figure 8:**
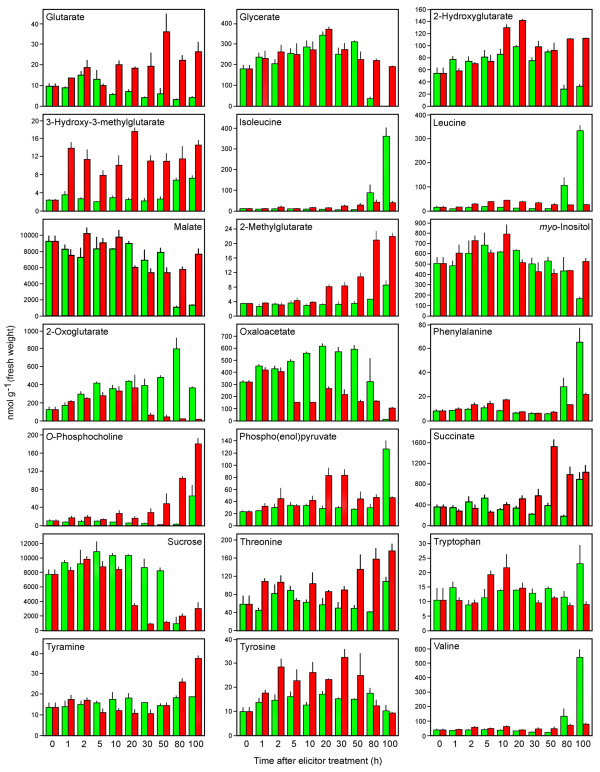
**Quantification of identified metabolites (glutarate to valine, alphabetically) in control (green) and elicitor-treated (red) opium poppy cell cultures at different time points post-elicitation**. Data are given as means ± SEM, which were calculated using three biological replicates. Quantification was achieved using Chenomx NMR Suite v. 4.6 with DSS as the internal standard.

A customized opium poppy NMR spectral library was created to identify and quantify individual metabolites [see Additional file 2]. A total of 212 compounds from diverse pathways are represented in the database, and were configured into a linkage map to reveal general metabolic relationships (Figure 6). A total of 42 compounds were conclusively identified and 102 known plant metabolites were unambiguously either below the analytical detection limit or were not present in the sample. The status of another 68 compounds could not be determined due to masking caused by the abundance of other metabolites. Figures 7 and 8 show the profiles of individual metabolites identified in control and elicitor-treated cells over the 100-h time course. Levels of carbohydrates including glucose, sucrose and fructose decreased more rapidly in elicitor-treated cells compared with controls. Glutarate and derivatives thereof were generally more abundant in elicitor-treated cells compared with controls.

Eleven amino acids were detected in control and elicitor-treated samples. The levels of most amino acids increased between 50 and 100 h in control cultures, but remained low in elicitor-treated cells. The amino acids glutamine and glutamate, which are involved in nitrogen metabolism, were generally lower in elicitor-treated cells relative to controls at time points after 5 h. Asparagine is also involved in nitrogen metabolism and generally showed higher levels 5 h after elicitor treatment, but overall was higher in 100-h control extracts. Tyrosine, the precursor to benzylisoquinoline alkaloids, increased in abundance between 1 and 50 h in elicitor-treated cells compared with controls. Tyramine levels were lower in elicitor-treated cells between 5 and 30 h, but were higher at 80 and 100 h compared with controls. Phenylalanine also increased from 2–10 h post-elicitation, but the largest cellular pools were detected in control cultures at 80 and 100 h. Two non-protein amino acids, GABA and β-alanine, showed a rapid accumulation in elicitor-treated cultures. It is notable that β-alanine was the only metabolite absent in control cultures and induced by elicitor treatment. Coumarate, an intermediate in phenylpropanoid metabolism and a derivative of phenylalanine, initially accumulated in both control and elicitor-treated cells, but decreased in abundance from 50–100 h. The increase in benzoate levels was more pronounced in elicitor-treated cells, reached a maximum at 50 h and thereafter declined gradually.

Erythrose 4-phosphate (E4P) and phosphoenolpyruvate (PEP) are precursors to the shikimate pathway. The abundance of E4P reflected the modulation of cellular carbohydrate pools. An initial increase in E4P levels in both control and elicitor-treated cells was followed by a more rapid decline in elicitor-treated cultures. In contrast, PEP levels remained relatively stable, but spiked 20 and 30 h post-elicitation and at 100 h in control cultures. Most tricarboxylic acid (TCA) cycle intermediates could be identified. Citrate levels increased in elicitor-treated cells and were higher at all time points compared with controls. In contrast, *cis*-aconitate pools were relatively similar and stable in control and elicitor-treated cells, but remained substantially higher in elicitor-treated cells at 80 and 100 h. Levels of 2-oxoglutarate gradually increased in both control and elicitor-treated cultures, but declined in elicitor-treated cells from 30–100 h. Succinate and fumarate levels were generally stable, but succinate pools were higher from 50–100 h in elicitor-treated cells. Oxaloacetate levels were lower in elicitor treated cells 5–80 h post-elicitation.

*O*-Phosphocholine, choline and ethanolamine are involved in phospholipid metabolism, however only *O*-phosphocholine levels increased in elicitor-treated cultures. In contrast, choline and ethanolamine showed spikes only late in the control time course. The level of caprylate, which is involved in fatty acid biosynthesis, increased and was marginally higher in elicitor-treated cells between 2- and 50 h post-elicitation.

## Discussion

The application of ^1^H NMR complements our previous attempt to deploy FT-ICR-MS to profile changes to the metabolome of opium poppy cell cultures in response to treatment with a fungal elicitor. Several interesting comparisons can be made. First, FT-ICR-MS provided quantitative information on 992 analytes, although only about 2% of these were identified based solely on comparison with available molecular mass data and corresponding molecular formulae [8]. In contrast, information was obtained for 70% of 212 target compounds using ^1^H NMR metabolomics coupled with Chenomx NMR Suite. The identification of compounds based on ^1^H NMR spectra is more reliable than the use of molecular mass, which only provides a molecular and not a structural formula. Proton NMR also revealed abundant cellular metabolites that were not detected by FT-ICR-MS. Notable among these were several amino acids, none of which were found in the extensive molecular mass database used in our previous study [8]. In contrast, alkaloid pathway intermediates and products, including *N*-methylcoclaurine, *N*-methylstylopine, protopine, dihydrosanguinarine and sanguinarine were identified by FT-ICR-MS [8]. However, no signature spectra for any alkaloids were detected using NMR. This is likely due to the poor solubility of these alkaloids in D_2_O. ^1^H NMR has proven effective and complementary to FT-ICR-MS for the compound-specific profiling of a plant cell metabolome.

The components in the elicitor preparation responsible for inducing the defence response in opium poppy cell cultures are not known. However, it has been hypothesized that fungal cell wall glucans are involved. Although we cannot rule out the possibility that minor changes in the detected metabolite profiles resulted from degradation of compounds in the fungal hydrolysate, dynamic and substantial modulations in the levels of numerous metabolites strongly supports genuine and profound changes in the plant cell metabolome.

Multivariate statistical analysis of spectral data provides a perspective on metabolome dynamics independent of the identification of individual metabolites. For example, PCA clusters datasets based on broad, unbiased relationships and provides clues about the general types of metabolites predominantly responsible for the variance among samples. Global metabolite fingerprinting of control and elicitor-treated opium poppy cell culture extracts revealed significant modulations in cellular metabolism within 5–10 h after the addition of the elicitor. Variations in the major peaks visible in the full-range ^1^H NMR spectra from key time points (i.e. 0, 5, 30 and 100 h) showed that resonances in the spectral region corresponding to carbohydrates decreased substantially in the 30 and 100 h samples from elicitor-treated cells, compared with controls (Figure 1). These data suggested that carbohydrates were consumed faster in cells treated with the elicitor relative to controls. The spectra of samples from elicitor-treated cells appear similar at 30 and 100 h. In contrast, samples from controls at 100 h display major differences in all spectral regions suggesting a broad metabolic reconfiguration likely due to the depletion of nutrients, especially sucrose, in the culture medium. The general similarity of the spectra for elicitor-treated cells at 30 and 100 h suggests that both anabolic and catabolic activities are substantially different in elicitor-treated cells compared with controls.

Global PCA showed that the metabolome of elicitor-treated cells was more dynamic than that of control cultures. Variance in the control samples was minimal at early points in the time course, but was substantial at 80 and 100 h (Figure 2). These data are in agreement with the PCA results of the relative abundance of 992 analytes from control and elicitor-treated opium poppy cell cultures detected by FT-ICR-MS [8]. OPLS-DA supported a clear separation between control and elicitor-treated cells in three distinct metabolic phases (i.e. 0–10 h, 20–50 h and 80 and 100 h) of the time course (Figures 3, 4 and 5). The supervised (OPLS-DA) analysis of these three phases allowed an examination of early (i.e. 0–10 hours) and intermediate (i.e. 20–50 hours) components of the defence response, in addition to sustained metabolic effects (i.e. 80 and 100 h). Interestingly, the most substantial differences between elicitor-treated and control cultures were detected at 80 and 100 hours after treatment. Although modulations in metabolite profile that occur more than 80 hours after elicitor treatment arguably do not represent specific elicitor-associated defence responses, the extended time course provides insight into the long-term consequences of environmental perturbations to the metabolome.

The corresponding loadings plots provided additional clues about the identity of metabolites predominantly responsible for the observed variance. Carbohydrates and organic acids contributed substantially to the separation between elicitor-treated and control cells up to 50 h post-elicitation. In contrast, amino acid levels were a major factor in the overall variance between control and elicitor-treated cells at 80 and 100 h. These results demonstrate the utility of metabolite fingerprint analysis, based on multivariate statistical approaches, in providing important clues about identity of metabolites that undergo substantial and differential modulations in abundance in control and elicitor-treated opium poppy cell cultures. However, quantitative, compound-specific profiling of the spectral data allowed an unprecedented examination of dynamic changes in the level of individual metabolites (Figures 7 and 8). An absolute quantification or information on relative abundance (i.e. either an absolute cellular concentration, or a reliable determination that the cellular pool size was below the analytical detection limit) was obtained for 144 cellular metabolites among a total of 212 diverse compounds in the customized opium poppy database [see Additional file 2].

### Carbohydrate metabolism

Sucrose plays a central role in plant metabolism and is a critical source of energy generation in plants. Pools of carbohydrates such as sucrose, glucose and fructose pools were depleted more rapidly in elicitor-treated cells than in controls, which reflects an increased requirement for carbon and energy to support secondary metabolism. An accelerated depletion of carbohydrate levels was also observed using FT-ICR-MS analysis of elicitor-treated opium poppy [8] and elicitor-treated *Medicago truncatula *[9] cell cultures. The abundance of several transcripts encoding pentose phosphate and glycolytic pathway enzymes also increased within 2 h after elicitor treatment of opium poppy cell cultures [8]. However, the availability of carbohydrate in elicitor-treated opium poppy cells does not appear to limit alkaloid production since the augmentation of carbohydrate in the culture medium has been reported not to improve sanguinarine accumulation [17]. A substantial demand on respiratory metabolism is also necessary to supply the precursors of the shikimate pathway, phosphoenolpyruvate (PEP) and erythrose 4-phosphate (E4P). Shikimate metabolism leads to the aromatic amino acids, of which tyrosine and phenylalanine are used as precursors for benzylisoquinoline alkaloid and phenylpropanoid metabolism in opium poppy. However, the levels of transcripts encoding phosphoglycerate mutase and enolase, which catalyze the last two glycolytic steps in PEP biosynthesis, were suppressed in elicitor-treated cells [8]. PEP is also derived from oxaloacetate by phosphoenolpyruvate carboxykinase (PEPCK) in gluconeogenesis. PEPCK transcript levels were also induced in elicitor-treated cells (K. Zulak and P. Facchini, unpublished results). Oxaloacetate levels were considerably lower in elicior-treated cells compared with controls; thus, it is possible that oxaloacetate is utilized for PEP synthesis in elicitor-treated cells. Gluconeogenesis was purportedly induced in maize embryos in response to pathogen challenge [18]. The activation of gluconeogenic pathways might explain the maintenance of carbohydrate pools in elicitor-treated cells. Moreover, gluconeogenic enolase was reportedly inhibited by 2-phosphoglycerate [19] the product of phosphoglycerate mutase. Transcript levels of phosphoglycerate mutase were suppressed in response to elicitor treatment in opium poppy cells [8].

In elicitor-treated parsley cells, the increased evolution of respiratory CO_2 _was accompanied by an induction in the levels of enzymes involved in glycolysis and the oxidative pentose phosphate pathway [20]. Although no intermediates between hexose sugars and PEP were identified, almost all intermediates in the TCA cycle were detected (Figures 6 and 7). Similar to FT-ICR-MS, no glycolysis or oxidative pentose phosphate pathway intermediates were identified using ^1^H-NMR, except for PEP. Several TCA intermediates, including succinate, malate and citrate became more abundant in elicitor-treated cells, compared with controls, as the time course progressed. Cellular pools of 2-oxoglutarate, which is involved in carbon/nitrogen sensing, also began to decrease more rapidly in elicitor-treated cells after 30 h.

The levels of almost every detected amino acid were significantly higher in controls relative to elicitor-treated cells at 80 and 100 h. At earlier time points, amino acids levels were marginally higher in elicitor-treated cells, suggesting a lower demand for nitrogen and/or increased proteolytic activity. Similarly, the elevated levels of choline and ethanolamine in control cultures at 80 and 100 h post-elicitation suggest less flux into fatty acid and lipid metabolism and/or enhanced phospholipid degradation compared with elicitor-treated cells. The levels of almost every glycolytic and TCA intermediate were also higher in elicitor-treated cells suggesting an increase in carbohydrate metabolism compared with control cultures. In control cells, induced catabolism might be necessary to provide energy to sustain respiration in response to sucrose starvation [21]. It is also possible that the catabolic pathways activated upon sucrose starvation at 80 and 100 h were suppressed in elicitor-treated cells to maintain flux into secondary metabolism.

### Nitrogen assimilation

Nitrogen assimilation in elicitor-treated opium poppy cell cultures has been reported to primarily involve NH_4_^+ ^[17]. In contrast, control cultures utilized NO_3_^- ^and NH_4_^+ ^equally. The major pathway involved in NH_4_^+^assimilation is the glutamine synthase/glutamine: α-oxoglutarate aminotransferase (GS/GOGAT) cycle. Glutamine and glutamate serve as nitrogen donors for the biosynthesis of compounds such as amino acids, nucleotides, chlorophylls, polyamines and alkaloids [22]. The GS/GOGAT cycle was also suggested to play a role in carbon/nitrogen sensing in plant cells [22]. However, transcripts for putatively plastidic (i.e. the closest homologue is plastid localized) GS and GOGAT are suppressed in elicitor-treated opium poppy cells [8], and glutamine and glutamate levels are lower in elicitor-treated cells relative to controls. In bean cell cultures treated with a fungal elicitor, GS activity decreased over a 24-h time course, but GOGAT activity was stable [23]. Both activities were stable in control bean cultures. In tobacco leaves challenged by a pathogen, treated with an elicitor or exposed to different phytohormones, nitrate reductase and choroplastic glutatmine synthase transcript levels and GS activity were suppressed. In contrast, cytosolic GS and glutamate dehydrogenase transcript levels and GDH activity were induced [24].

The down-regulation of the GS/GOGAT cycle in elicitor-treated opium poppy cells raises the question: how is nitrogen assimilated and stored for the massive demands of alkaloid biosynthesis? In some species asparagine rather than glutamine is preferred for the transport and/or storage of nitrogen. In elicitor-treated opium poppy cells, asparagine increased in abundance later than most amino acids. The concentration of asparagine also increased in *Pseudomonas syringae*-infected tomato leaves, suggesting that asparagine, and not glutamine is primarily involved in the transport of nitrogen to healthy parts of the plant [25].

### Phospholipid metabolism

Phospholipids play several roles in cellular function including signal transduction, membrane trafficking and cytoskeletal rearrangement, and have also been implicated in the hypersensitive response and systemic acquired resistance [26]. Phospholipase A_2 _(PLA_2_) hydrolyzes phospholipids such as phosphatidylcholine (PC) into a lysophospholipid (lysoPC) and a fatty acid [27]. PLA_2 _activity was induced in *Botrytis cinerea*-infected tobacco leaves, compared with controls, but not in response to drought, wounding, reactive oxygen intermediates, salicylic acid or MeJA [28]. This suggests that PLA_2 _induction is specifically associated with pathogen challenge and not to a general stress response. In parsley and tobacco cell cultures, PLA_2 _was also induced in response to elicitor treatment [29]. In opium poppy cells, only *O*-phosphocholine increased in response to elicitor treatment. The first step in choline biosynthesis involves the decarboxylation of serine to ethanolamine [30]. Choline biosynthesis can follow three parallel pathways each catalyzed by the action of *N*-methyltransferases on free-bases [31], phospho-bases [32] or phosphatidyl-bases [33]. Since phosphocholine can be incorporated directly into phosphatidylcholine [33], the substrate for PLA_2_, the relatively low abundance of cellular phosphocholine pools early in the time course might reflect increased flux through the phosphatidyl-base pathway to lysoPC, which has been implicated in pH signaling in elicitor-treated *Eschscholzia californica *cells [34]. PLA_2 _has also been reported to play an important role in the production of linolenic acid, the precursor to jasmonic acid (JA), in response to stress [35]. It is notable that phosphatidyl choline, linolenic acid and (+)-7-jasmonic acid were identified in elicitor-treated opium poppy cells using FT-ICR-MS [8]. These data suggest that JA signaling is an important component of the defence response in opium poppy cells.

### Non-protein amino acids

GABA is a ubiquitous non-protein amino acid synthesized from glutamate by glutamate decarboxylase (GAD) in a pathway known as the GABA shunt that bypasses several steps of the TCA cycle. GABA is converted to succinate semialdehyde by GABA transaminase and then oxidized to succinate by succinic semialdehyde dehydrogenase. In plants, GABA generally accumulates in response to biotic and abiotic stresses [36]. GABA levels increased in response to both MeJA and yeast elicitor in *M. truncatula *cell cultures [9]. In opium poppy cultures, cellular pools of GABA increased to maximum levels between 10 and 50 h after elicitor treatment. Plant GAD is regulated by Ca^2+^/calmoldulin [37,38] cytoplasmic acidification [39] and glutamate availability [40]. GABA accumulation was reported to correlate with an inhibition in the conversion of glutamate to glutamine, suggesting a role for GABA in stress responses as a temporary nitrogen store [41]. GABA and glutamate levels were also linked to diurnal rhythms, suggesting that GABA might buffer glutamate content and contribute to carbon/nitrogen balance [42]. GABA could replace glutamine as a temporary nitrogen store in opium poppy cultures and might also participate in carbon/nitrogen signaling. Although the latter process is not well understood, the lack of a GABA gradient in Arabidopsis pistils was implicated in the misguidance of pollen tubes suggesting a role for GABA in intercellular signaling [43].

β-Alanine is a non-protein amino acid synthesized mainly by polyamine (i.e. spermine and spermidine) degradation and involved in coenzyme A (CoA) biosynthesis via pantothenate [44,45], uracil [46] or possibly from propionate [47]. In opium poppy cells, β-alanine was only detected in elicitor-treated cultures suggesting a role in the defence response. β-Alanine also accumulated in MeJA-treated *M. truncatula *cells [9]. The induction of β-alanine accumulation could reflect an increase in CoA biosynthesis. CoA is a ubiquitous metabolite that is involved in the oxidation of fatty acids, carbohydrates and amino acids, and plays a key role in the biosynthesis of many secondary metabolites including phenylpropanoids.

### Shikimate and aromatic compounds

The shikimate pathway begins with the condensation of E4P and PEP, and links carbohydrate metabolism with aromatic amino acids and derivatives in plants and microorganisms through the formation of chorismate [48]. Although E4P and PEP were identified in opium poppy cell cultures (Figures 7 and 8), no shikimate pathway intermediates were detected. The level of transcripts encoding each enzyme in the shikimate pathway increased in elicitor-treated opium poppy cells as early as 1–2 h post-treatment [8]. The abundance profile of E4P was similar to those of carbohydrates (i.e. an initial increase followed by a more rapid decrease in elicitor-treated cells compared with controls) possibly due to its metabolic link to glucose, fructose and sucrose (Figure 6). In contrast, PEP showed a brief peak in abundance 20–30 h post-elicitation, and increased levels at 100 h in control cultures.

Phenylpropanoids are induced in response to many stresses including UV, pathogen challenge, wounding, low temperature and nutrient deficiency [49]. Levels of phenylalanine, the precursor to phenylpropanoids, initially increased in elicitor-treated cells, which correlates with the induction of phenylalanine ammonia lyase (*PAL*) transcripts within 2 h post-elicitiation [8]. Two phenylalanine derivatives, benzoic acid (BA) and coumarate, were also detected. BA is mainly derived from phenylalanine, but the synthesis of BA and salicylic acid via isochorismate has also been demonstrated in Arabidopsis [50]. BA was induced in tobacco mosaic virus (TMV) infected tobacco plants undergoing the hypersensitive response and tobacco cell cultures elicited with β-megaspermin from *Phytophtora megasperma *[51]. BA and its derivatives play important roles in biotic and abiotic stress responses and are incorporated into several secondary defence-related metabolites [52]. For example, methylbenzoate was inducible in Arabidopsis leaves challenged with various biotic and abiotic stresses [53] and benzoid carboxymethyltransferases were induced under similar conditions [54]. Similar compounds have not yet been identified in opium poppy. Coumarate levels initially increased in elicitor-treated cells more rapidly than in controls, but subsequently decreased from 50–80 h in both cases. Coumarate is synthesized from phenylalanine via the successive actions of PAL and cinnamate 4-hydroxylase (C4H). In opium poppy, *PAL *and *C4H *transcript levels were induced in response to elicitor treatment, but returned to basal levels within 100 h [8]. Three additional phenylpropanoids, ferulate, 5-hydroxyferulic acid, and coumaroyl shikimate were identified in elicitor-treated opium poppy cell cultures using FT-ICR-MS, [8]. Ferulate is hydroxylated to 5-hydroxyferulic acid, which is then methylated to form sinapate. Cinnamate and BA derivatives were reportedly incorporated into the cell wall fraction of *Musa acuminata *roots in response to *Fusarium oxisporum *elicitors [55]; thus, BA and sinapate derivatives might also be incorporated into opium poppy cell walls as part of the overall defence response.

Tyrosine and tyramine are precursors to both benzylisoquinoline alkaloid and hydroxycinnamic acid amide metabolism in opium poppy. Tyrosine/DOPA decarboxylase (TYDC), which converts tyrosine and DOPA to tyramine and dopamine, respectively, was rapidly induced upon elicitation [56]. Tyramine hydroxycinnamoyl CoA: tyramine hydroxycinnamoyltransferase (THT) condenses tyramine and hydroxycinnamoyl-CoA esters to form hydroxycinnamic acid amides and is induced in response to elicitor treatment [57]. Cellular tyrosine pools increased in elicitor-treated between 2–50 h, but tyramine levels were similar or lower relative to controls until 80 and 100 h post-elicitation (Figure 8). The initial increase in tyrosine levels in elicitor-treated cells might reflect the role of this amino acid as a precursor for both amide and alkaloid biosynthesis.

## Conclusion

Metabolite profiling by ^1^H NMR is a useful tool to characterize the metabolic response of plant cell cultures to environmental perturbations, such as elicitor treatment [8,9]. An impressive 70% success rate in the assignment of an absolute or relative quantification to 212 target compounds in the opium poppy cell culture metabolome was achieved. The identification of additional metabolites will require the fractionation of cellular extracts to reduce masking by abundant metabolites, and the addition of reference compounds to the signature spectra database. Such refinements are feasible and should encourage further development of the still untapped potential of ^1^H NMR metabolomics and targeted profiling.

The metabolic demands of the defence response in elicitor-treated opium poppy cell cultures involves the coordinate transcriptional induction of key components of both primary and secondary pathways [8]. Our results show that the induction of alkaloid and other secondary and defence pathways in response to environmental perturbations is accompanied by the extensive reprogramming of specific primary metabolic networks. The availability of broad-scope metabolomics and transcriptomics databases will facilitate the establishment of a systems biology approach to discover biological components and processes involved in the formation of benzylisoquinoline alkaloids and other secondary metabolites in opium poppy. The extensive integration of plant metabolic networks revealed by metabolomics demonstrates the importance of establishing a comprehensive model to predict the consequences of perturbations in secondary metabolism on the regulation of primary pathways. Predictive metabolic engineering of alkaloid biosynthesis in opium poppy should benefit from rational adjustments to the flux of the upstream metabolic pathways that provide precursors and cofactors necessary for the assembly of desired natural products.

## Methods

### Cell cultures and elicitation

Opium poppy (*Papaver somniferum *cv. Marianne) cell suspension cultures were maintained under fluorescent lights at 23°C on Gambourg 1B5C medium consisting of B5 salts and vitamins, 100 mg L^-1 ^*myo*-inositol, 1 g L^-1 ^hydrolyzed casein, 20 g L^-1 ^sucrose, and 1 mg L^-1^, and 1 mg L^-1 ^2,4-dichlorophenoxyacetic acid (2,4-D). Cells were sub-cultured every 6 d using a 1:3 dilution of inoculum to fresh medium. Fungal elicitors were prepared according to [58]. Sections (1 cm^2^) of *Botrytis cinerea *mycelia grown on potato dextrose agar were used to inoculate 50 mL of 1B5C medium including supplements, but lacking 2,4-D. Mycelium cultures of *B. cinerea *were grown at 120 rpm on a gyratory shaker at 23°C in the dark for 6 d. Mycelia and medium were homogenized and autoclaved at 121°C for 20 min. One milliliter of the fungal homogenate was added to 50 mL of cultured cells in rapid growth phase (2–3 d after subculture). Cells were collected by vacuum filtration at different time points after elicitor treatment. Control cultures (i.e. not treated with the elicitor) were also collected at each time point. All samples were stored at -80°C until used.

### Metabolite extraction

Frozen cell culture tissue (0.75 g) was ground to a fine powder under liquid nitrogen with a mortar and pestle and extracted in three 10-mL aliquots of 80% (v/v) ethanol. Aliquots were pooled and centrifuged for 10 min to pellet cell debris. The supernatant was lyophilized in a vacuum centrifuge at ambient temperature, re-dissolved in 5 mL H_2_O, de-ionized twice with 1 mL Chelex-100 Resin (Biorad, Hercules, CA), and re-lyophilized. Samples were re-dissolved in D_2_O containing 100 mM KD_2_PO_4_, pH 7.000 ± 0.002, 10 mM NaN_3_, and 0.5 mM 2,2-dimethyl-2-silapentane-5-sulfonate (DSS) as an internal standard.

### NMR Spectroscopy

^1^H NMR spectra were acquired using the standard Bruker noesypr1d pulse sequence in which the residual water peak was irradiated during the relaxation delay of 1.0 s and during the mixing time of 100 ms. All experiments were performed on a Bruker Advance 600 spectrometer (Bruker Biospin, Inc., Milton, Canada) operating at 600.22 MHz and equipped with a 5 mm TXI probe at 298°K. A total of 256 scans were collected into 65,536 data points over a spectral width of 12,195 Hz, with a 5 s repetition time. A line broadening of 0.5 Hz was applied to the spectra prior to Fourier transformation, phasing and baseline correction. Additional NMR experiments performed to confirm chemical shift assignments included total correlation spectroscopy (TOCSY) and heteronuclear single quantum coherence spectroscopy (HSQC), using standard Bruker pulse programs.

### Data analysis

Identification and quantification of individual metabolites was performed using the Profiler module of the Chenomx NMR Suite v.4.6 (Chenomx. Inc., Edmonton, Canada). ^1^H NMR spectra were compared against a library containing 212 plant-specific compounds. This library contains the unique ^1^H NMR spectra of each standard compound recorded at 600 MHz quantified by the addition of a known amount of DSS, which also served as a chemical shift indicator. For the purposes of this study, one-dimensional ^1^H NMR signatures corresponding to selected compounds not present in the standard Chenomx library, including those of several benzylisoquinoline alkaloids, were used to create a custom opium poppy database [see Additional file 2]. Comparisons of NMR spectra with this database produced a list of compounds and their respective concentrations. After excluding all shifts related to the solvent (i.e. in the range of 4.5–5.0 ppm) and DSS, the remaining spectral regions were divided into 0.04-ppm bins. The bins were normalized to the area under the DSS peak to assess the contribution of individual metabolites to the spectrum as well as total spectral area to correct for dilution effects. Chemometric analysis was performed using SIMCA-P v.11.5 (Umetrics, Inc., Kinnelon, NJ) using either unsupervised principal component analysis (PCA) or supervised orthogonal partial least square discriminate analysis (OPLS-DA) [59]. OPLS-DA is a supervised analysis tool that was used on three time-course regions (i.e. 0–10 h, 20–50 h, and 80–100 h post elicitation) to reveal differences in the metabolite profiles otherwise masked by PCA using all data points [59]. OPLS-DA allows for focus on variance due to elicitation alone while minimizing other biological or analytical variables. For both PCA and OPLS-DA, spectral regions were the X-matrix. All X-variables were *pareto *scaled to minimize the influence of baseline deviations and noise. For OPLS-DA, class difference (e.g. control versus elicitor-treated) was the Y-matrix. The quality of each model was determined by the goodness of fit parameter (R^2^) and the goodness of prediction parameter based on the fraction correctly predicted in a 1/7 cross-validation (Q^2^).

## List of abbreviations

DSS, 2,2-dimethyl-2-silapentane-5-sulfonate; FT-ICR-MS, Fourier transform ion cylotron resonance-mass spectrometry; ^1^H NMR, proton-nuclear magnetic resonance mass spectroscopy; OPLS-DA, orthogonal partial least-squares-discriminant analysis; PCA, principal component analysis.

## Authors' contributions

KZ conceived the experimental design, performed the sample preparation and data analysis, and wrote the first draft of the manuscript. AW supervised and assisted with the bioinformatics and statistical analyses. HV supervised the NMR spectroscopy. PF conceived of the study, prepared the figures and finalized the manuscript.

## Supplementary Material

Additional file 1 Bin numbers used in PCA and OPLS-DA, the regions of the spectra they represent and compounds present within those regions. Variable importance numbers pertain only to OPLS-DA and larger numbers indicate a greater contribution of that bin to observed variance between control and elicited cells. Click here for file

Additional file 2 List of metabolites for which one-dimensional ^1^H NMR signatures are available in a Chenomx NMR Suite compound database customized for opium poppy. An asterisk denotes a metabolite not present in the standard Chenomx library, but added to the customized database. Click here for file
