# Supplementary material for: Quantitative 1H NMR metabolomics reveals extensive metabolic reprogramming of primary and secondary metabolism in elicitor-treated opium poppy cell cultures
Source: BMC Plant Biol. 2008 Jan 22;8:5. doi: 10.1186/1471-2229-8-5 (PMC2257952; doi:10.1186/1471-2229-8-5)
Supplement: Additional file 1 — Bin numbers used in PCA and OPLS-DA, the regions of the spectra they represent and compounds present within those regions. Variable importance numbers pertain only to OPLS-DA and larger numbers indicate a greater contribution of that bin to observed variance between control and elicited cells. [file 1471-2229-8-5-S1.PDF]

Additional file 1: Bin numbers used in PCA and OPLS-DA, the regions of the spectra they represent and compounds present within those regions. Variable importance numbers pertain only to OPLS-DA and larger numbers indicate a greater contribution of that bin to observed variance between control and elicited cells.

Bin	Range (ppm)	Metabolites Present in Range	0-10 h	Variable Importance	Coordinate	20-50 h	Variable Importance	Coordinate	80-100 h	Variable Importance	Coordinate
5	0.85-0.89	Pantothenate, Unknown	X	1.82032	0.128318, -0.04715	X	1.01734	0.069061, 0.009883			
6	0.89-0.93	Isoleucine, Pantothenate	X	1.01654	0.074846, -0.01032				X	1.54398	-0.10264, -0.0966
7	0.93-0.97	Leucine							X	2.49573	-0.16742, -0.13783
8	0.97-1.01	Valine, Isoleucine							X	2.28699	-0.15198, -0.14532
9	1.01-1.05	Valine, 2-Methylglutarate							X	1.1724	-0.07715, -0.13489
13	1.17-1.21	Unknown	X	1.27332	0.098731, 0.074852	X	1.67807	0.112188, 0.097408	X	1.88365	0.124986, -0.04054
14	1.21-1.25	Unknown	X	1.17348	0.086563, -0.02561	X	1.08305	0.073114, 0.049555			
15	1.25-1.29	Caprylate	X	3.34895	0.232869, -0.10445	X	1.20382	0.083444, -0.1914	X	1.10778	-0.07025, -0.04149
16	1.29-1.33	Threonine	X	3.8643	0.265454, -0.04825	X	2.39451	0.160665, 0.057251			
17	1.33-1.37	Unknown	X	2.24785	0.165511, 0.025473	X	1.93677	0.130046, 0.061882	X	1.99509	0.132112, -0.03633
20	1.45-1.49	Alanine	X	1.1938	0.066437, 0.016213						
22	1.53-1.57	Caprylate	X	1.23484	0.084121, -0.04409						
23	1.57-1.61	Unknown	X	1.23422	0.085558, -0.04163						
26	1.69-1.73	Leucine, Unknown							X	1.22735	-0.08074, -0.09112
30	1.85-1.89	4-Aminobutyrate	X	1.56351	0.105678, 0.036819	X	1.42217	0.094611, 0.094422			
31	1.89-1.93	Acetate, 4-Aminobutyrate	X	1.48604	0.098731, 0.179595	X	1.85832	0.123636, 0.178514			
33	1.97-2.01	2-Hydroxyglutarate	X	1.27475	0.088358, 0.01133	X	1.02380	0.069092, -0.01275			
34	2.01-2.05	Glutamate	X	1.66497	0.113511, -0.00427	X	1.23455	0.082915, 0.031832	X	1.29234	-0.08715, -0.09516
35	2.05-2.09	Glutamate							X	1.23282	-0.08348, -0.08909
36	2.09-2.13	Glutamate, Unknown							X	1.80002	-0.12309, -0.1073
37	2.13-2.17	Glutamate, Glutarate							X	1.33356	-0.09104, -0.09485
39	2.21-2.25	Unknown	X	1.77704	0.119393, -0.00702						
40	2.25-2.29	*myo*-Inositol	X	2.09231	0.14291, 0.049715	X	1.96120	0.130386, 0.158694			
41	2.29-2.33	4-Aminobutyrate	X	1.01073	0.065748, 0.087765	X	1.40530	0.093723, 0.10196			
42	2.33-2.37	Malate, Glutamate				X	1.89810	0.127711, 0.151631	X	1.20139	0.078895, -0.17135
43	2.37-2.41	Malate, Succinate				X	2.51149	0.165939, 0.367149	X	1.35919	-0.09041, -0.2326
44	2.41-2.45	2-Oxoglutarate, Glutamine, 3-Hydroxy-3-methylglutarate							X	3.14312	-0.21162, -0.05173
46	2.49-2.53	Citrate	X	2.36282	0.153598, 0.248386	X	2.08612	0.142422, 0.107433	X	3.08294	0.207316, -0.02376
47	2.53-2.57	Citrate, Malate	X	2.26392	0.146047, 0.196233	X	2.11153	0.144195, 0.166075	X	3.32557	0.222959, -0.01967
49	2.61-2.65	Citrate, Malate	X	3.15313	0.205797, 0.426246	X	2.63987	0.180243, 0.031634	X	4.66375	0.314543, -0.02764
50	2.65-2.69	Malate	X	1.82779	0.117904, 0.361764	X	2.14689	0.146736, 0.235717	X	2.23116	0.149212, -0.12758
51	2.69-2.73	Aspartate, Unknown							X	1.40074	-0.09192, -0.06709
52	2.73-2.77	Unknown	X	1.05466	0.070127, -0.03385						
57	2.93-2.97	Asparagine, Tyramine							X	1.14296	-0.07374, -0.1077
58	2.97-3.01	4-Aminobutyrate	X	1.91088	0.133355, 0.007892	X	1.78792	0.118733, 0.092399	X	2.21903	-0.14982, -0.03841
59	3.01-3.05	4-Aminobutyrate	X	1.07874	0.069531, 0.033221	X	1.05956	0.070234, 0.080162			
61	3.09-3.13	*cis*-Aconitate, Phenylalanine, Unknown				X	1.06690	0.07092, 0.122361			
62	3.13-3.17	Ethanolamine, Unknown							X	1.00795	-0.06858, -0.06819
63	3.17-3.21	Choline, Tyrosine, Unknown							X	3.71456	-0.24975, -0.11715
64	3.21-3.25	Glucose, Unknown	X	1.57790	-0.10175, -0.17125	X	1.09523	-0.07316, -0.09168			
65	3.25-3.29	*myo*-Inositol				X	1.04119	0.068794, 0.124744			
66	3.29-3.33	Unknown				X	1.24539	0.081924, 0.134755	X	1.03585	0.069905, -0.08005
68	3.37-3.41	Glucose	X	2.25971	-0.14731, -0.15858	X	1.69306	-0.11253, -0.15087			
69	3.41-3.45	Sucrose, Glucose	X	1.90493	-0.12791, -0.07195	X	1.39514	-0.0934, -0.15865			
70	3.45-3.49	Sucrose, Glucose	X	3.58210	-0.22977, -0.12038	X	2.73407	-0.18324, -0.32388	X	1.00922	0.068785, 0.170836
71	3.49-3.53	Citrate	X	1.85009	-0.11332, 0.020631				X	2.27015	-0.15318, -0.02136
72	3.53-3.57	Sucrose, Fructose	X	2.94168	-0.20072, -0.2491	X	3.20463	-0.21536, -0.20291	X	1.35377	0.091023, 0.28009
73	3.57-3.61	Fructose, Threonine, *myo*-Inositol, Unknown				X	1.08378	0.071513, 0.336302	X	2.23133	-0.14961, -0.0294
74	3.61-3.65	*myo*-Inositol				X	1.89920	0.125816, 0.394436			
75	3.65-3.69	Sucrose, Glucose, Fructose	X	3.61697	-0.23489, -0.14737	X	3.49981	-0.2351, -0.3617	X	2.16338	0.143971, 0.246988
76	3.69-3.73	Fructose, Glucose	X	2.72750	-0.17361, -0.35293	X	2.62036	-0.17581, 0.02798			
77	3.73-3.77	Sucrose, Glucose, Glutamine	X	2.51694	-0.17683, -0.04873	X	1.90167	-0.12888, -0.12559	X	1.30811	-0.09591, -0.02071
78	3.77-3.81	Sucrose, Fructose	X	3.92417	-0.26043, -0.22079	X	4.31271	-0.28873, -0.40558	X	1.66665	0.111343, 0.315102
79	3.81-3.85	Sucrose, Glucose, Fructose	X	3.12179	-0.23068, -0.19442	X	4.23298	-0.28477, -0.35313	X	2.77962	0.184591, 0.254662
80	3.85-3.89	Sucrose, Glucose, Fructose	X	2.41562	-0.16324, -0.25236	X	2.80575	-0.18836, -0.1706			
81	3.89-3.93	Sucrose, Glucose, Fructose	X	1.51470	-0.09733, -0.15808	X	1.39491	-0.09484, 0.124898	X	1.16282	-0.08085, 0.112453
82	3.93-3.97	Unknown				X	1.38885	0.091918, 0.250282			
83	3.97-4.01	Fructose	X	1.82052	-0.11002, -0.30267	X	1.86360	-0.12542, 0.107535			
84	4.01-4.05	Sucrose, Fructose	X	2.12951	-0.1387, -0.13716	X	2.38210	-0.16029, -0.22617	X	1.04985	0.069289, 0.192874
88	4.17-4.21	Sucrose				X	1.67018	-0.11223, -0.23212	X	1.71548	0.114059, 0.067822
89	4.21-4.25	Sucrose				X	1.43597	-0.09667, -0.2042	X	1.42189	0.094, 0.048312
90	4.25-4.29	Malate	X	1.28003	0.072589, 0.231818	X	1.04267	0.071364, 0.172298	X	2.60382	0.173253, -0.02542
91	4.29-4.33	Malate	X	1.16903	0.066575, 0.211728				X	1.66695	0.109346, -0.04753
116	5.29-5.33	Unknown	X	1.48308	0.100437, -0.02758						
118	5.37-5.41	Sucrose	X	1.59892	-0.11782, -0.01386	X	2.45486	-0.1645, -0.31295	X	2.10400	0.140526, 0.108395
124	5.61-5.65	Unknown							X	1.01005	-0.06806, -0.01569
125	5.65-5.69	Unknown							X	1.61228	-0.10859, -0.01966
145	6.45-6.49	Unknown							X	1.08481	-0.07343, -0.02979
146	6.49-6.53	Fumarate, Unknown							X	1.04763	-0.06991, -0.05082
156	6.89-6.93	Tyramine							X	1.88129	-0.12741, -0.04166
157	6.93-6.97	Unknown	X	1.02870	0.069819, 0.025087				X	1.70098	-0.115, -0.02338
163	7.17-7.21	Tyramine							X	1.24946	-0.08414, -0.03586
173	7.57-7.61	Glutamine							X	2.26265	-0.15245, -0.03783
174	7.61-7.65	Unknown							X	1.33936	-0.0903, -0.01867
179	7.81-7.85	Unknown							X	1.42209	-0.09536, -0.01643
194	8.41-8.45	Formate, Unknown							X	1.11346	0.074205, -0.01488
