# Supplementary material for: Quantitative 1H NMR metabolomics reveals extensive metabolic reprogramming of primary and secondary metabolism in elicitor-treated opium poppy cell cultures
Source: BMC Plant Biol. 2008 Jan 22;8:5. doi: 10.1186/1471-2229-8-5 (PMC2257952; doi:10.1186/1471-2229-8-5)
Supplement: Additional file 2 — List of metabolites for which one-dimensional 1H NMR signatures are available in a Chenomx NMR Suite compound database customized for opium poppy. An asterisk denotes a metabolite not present in the standard Chenomx library, but added to the customized database. [file 1471-2229-8-5-S2.PDF]

Additional file 2: List of metabolites for which one-dimensional ^{1}H NMR signatures are available in a Chenomx NMR Suite compound database customized for opium poppy. An asterisk denotes a metabolite not present in the standard Chenomx library, but added to the customized database.

1. Acetaldehyde
2. Acetamide
3. Acetate
4. Acetoacetate
5. Acetone
6. *N*-Acetylasparate
7. *N*-Acetylglutamate
8. Acetylsalicylate
9. *N*-Acetyltyrosine
10. *cis*-Aconitate
11. *trans*-Aconitate
12. Adenine
13. Adenosine
14. Adenosine diphosphate *
15. Adenosine triphosphate *
16. *S*-Adenosylhomocysteine
17. *S*-Adenosylmethionine *
18. Alanine
19. β-Alanine
20. Alloisoleucine
21. 2-Aminobutyrate
22. 4-Aminobutyrate
23. 3-Aminoisobutyrate
24. Anserine
25. Arginine
26. Asparagine
27. Aspartate
28. Benzoate
29. Betaine
30. Biotin
31. Butanone
32. Butyrate
33. Caffeate *
34. Caprate
35. Caprylate
36. *N*-Carbamoylaspartate
37. *N*-Carbamoyl-ß-alanine
38. Cholate
39. Choline
40. Cinnamate *
41. Citrate
42. Citrulline
43. Codeine *
44. Coumarate *
45. Cysteine
46. Cystine
47. Cytosine
48. 2’-Deoxyadenosine
49. 2’-Deoxyguanosine
50. 2’-Deoxyinosine
51. 1,3-Dihydroxyacetone
52. 5,6-Dihydrothymine
53. 5,6-Dihydrouracil
54. Dihydroxyacetone phosphate *
55. Dimethylamine
56. *N,N*-Dimethylformamide
57. *N,N*-Dimethylglycine
58. 1,7-Dimethylxanthine
59. L-DOPA *
60. Dopamine *
61. Erythrose-4-phosphate *
62. Ethanol
63. Ethanolamine
64. Ethylene glycol
65. Ethylmalonate
66. Ferulate
67. Formate
68. Fructose
69. Fructose-1,6-bisphosphate *
70. Fructose-6-phosphate *

71. Fucose
72. Fumarate
73. 2-Furoate
74. Galactarate
75. Galactonate
76. Galactose
77. Glucarate
78. Glucitol
79. Gluconate
80. Glucose
81. Glucose-6-phosphate *
82. Glutamate
83. Glutamine
84. γ-Glutamylphenylalanine
85. Glutarate
86. Glutaric acid monomethyl ester
87. Glutathione
88. Glycerate
89. Glycerol
90. Glycine
91. Glycolate
92. Glycylproline
93. Guanidoacetate
94. Histadine
95. Homocitrulline
96. Homocysteine
97. Homoserine
98. Homovanillate
99. *trans*-4-Hydroxy-L-proline
100. 4-Hydroxybenzoate
101. 2-Hydroxybutyrate
102. 3-Hydroxybutyrate
103. 4-Hydroxybutyrate
104. 2-Hydroxyglutarate
105. 3-Hydroxyisovalerate
106. 5-Hydroxyindole-3-acetate
107. 5-Hydroxylysine
108. 3-Hydroxy-3-methylglutarate
109. 4-Hydroxy-3-methoxymandelate
110. 2-Hydroxy-3-methylvalerate
111. 3-Hydroxyphenylacetate
112. 4-Hydroxyphenylacetate
113. 2-Hydroxyvalerate
114. Hypoxanthine
115. Imidazole
116. Indole-3-acetate
117. Inosine
118. *myo*-Inositol
119. Isobutyrate
120. Isocaproate
121. Isocitrate
122. Isoeugenol
123. Isoleucine
124. Isopropanol
125. Isovalerate
126. *N*-Isovaleroylglycine
127. Lactate
128. Lactose
129. Leucine
130. Lysine
131. Malate
132. Maleate
133. Malonate
134. Mannitol
135. Mannose
136. Methanol
137. Methionine
138. Methylamine
139. 2-Methylglutarate
140. 3-Methylglutarate
141. Methylguanidine
142. π-Methylhistadine
143. τ-Methylhistadine
144. Methylmalonate
145. 3-Methyl-2-oxovalerate
146. 3-Methylxanthine
147. 5-Methoxysalicylate
148. Methylsuccinate
149. Morphine *
150. NAD^+
151. Noscapine *
152. Ornithine
153. Oxaloacetate
154. 2-Oxobutyrate
155. 2-Oxoglutarate
156. 2-Oxovalerate

157. Panthothenate
158. Papaverine
159. Phenol
160. Phenylacetate
161. Phenylacetylglycine
162. Phenylalanine
163. 3-Phenylproprionate
164. *O*-Phosphocholine
165. Phosphoenolpyruvate *
166. *O*-Phosphoethanolamine
167. 6-Phosphogluconic acid *
168. 2-Phosphoglycerate
169. 2-Phenylproprionate
170. 3-Phosphoserine *
171. *O*-Phosphoserine
172. Phthalate
173. Proline
174. Proprionate
175. Propylene glycol
176. Pyridoxine
177. 4-Pyroxidate
178. Pyruvate
179. Quinolinate
180. *S*-Reticuline *
181. Riboflavin
182. Ribose
183. Ribose-5-phosphate *
184. Salicylate
185. Sanguinarine *
186. Serine
187. Shikimate *
188. Stylopine *
189. Suberate
190. Succinate
191. Succinylacetone
192. Sucrose
193. *S*-Sulfocysteine
194. Tartrate
195. Tetrahydrofolic acid *
196. Thebaine *
197. Threonate
198. Threonine
199. Thymine
200. Trimethylamine
201. Trimethylamine-*N*-oxide
202. Tryptophan
203. Tyramine *
204. Tyrosine
205. Uracil
206. Uridine
207. Valerate
208. Valine
209. Vanillate
210. Xanthine
211. Xanthosine
212. Xylose
